# Supplementary material for: Systematic Review with Trial Sequential Analysis of Prophylactic Antibiotics for Acute Pancreatitis
Source: Antibiotics (Basel). 2022 Sep 3;11(9):1191. doi: 10.3390/antibiotics11091191 (PMC9495153; doi:10.3390/antibiotics11091191)
Supplement: Supplementary file 1 [file antibiotics-11-01191-s001.zip › Supplementary Figure S1.pdf]

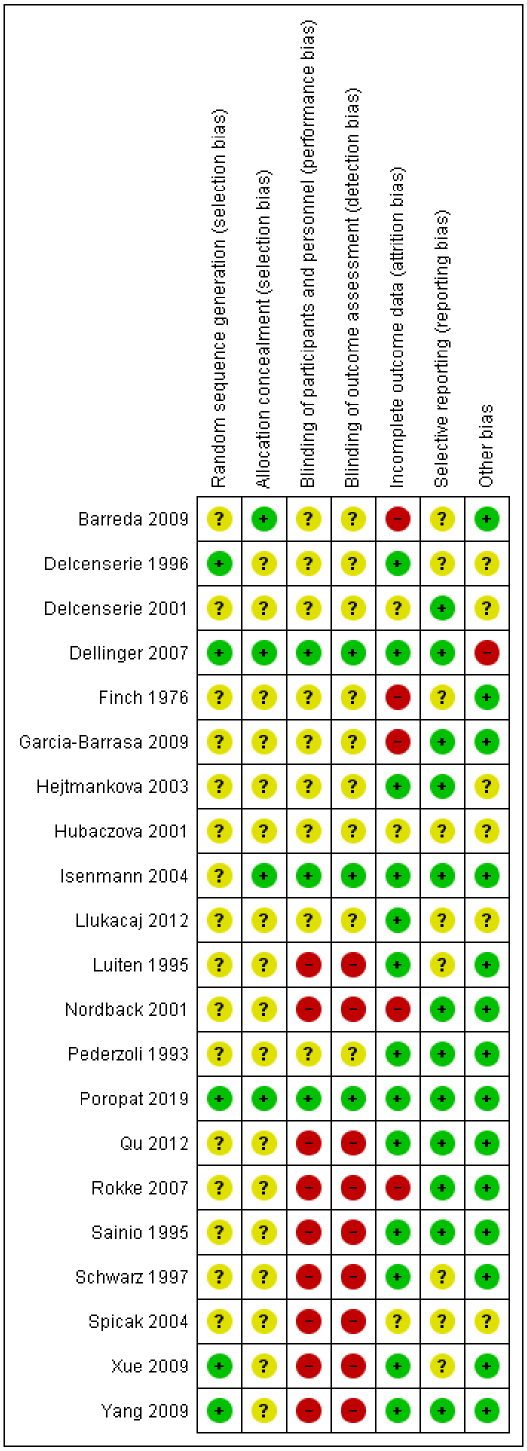

Figure S1. Risk of bias summary: review authors' judgements about each risk of bias item for each included study, the green circle indicating low risk of bias, yellow indicating unclear risk of bias, and red circle indicating high risk of bias.
