# Supplementary material for: Systematic Review with Trial Sequential Analysis of Prophylactic Antibiotics for Acute Pancreatitis
Source: Antibiotics (Basel). 2022 Sep 3;11(9):1191. doi: 10.3390/antibiotics11091191 (PMC9495153; doi:10.3390/antibiotics11091191)
Supplement: Supplementary file 1 [file antibiotics-11-01191-s001.zip › Supplementary Table S1.pdf]

Table S1. Characteristics of included studies.

Study	Year of publication	Country of origin	Antibiotic group, n (gender, age)	Control group, n (gender, age)	Etiology of AP	Type of intervention	Type of control	Risk of bias
Barreda L, et al.	2009	Peru	24 (37% females, median age 49, range 15-80)	34 (44% females, median age 51, range 15-76)	N/A	Imipenem 500 mg i.v. every 6 h for 14 days	No intervention	High
Delcenserie R, et al.	1996	France	11 (9% females, mean age (SD) = 43.9±10.5)	12 (8% females, mean age (SD) = 41.7±12.6)	N/A	Ceftazidime 2 g i.v. every 8 h; amikacin 7.5 mg/kg every 12 h; metronidazole 0.5 g every 8 h for 10 days	No intervention	High
Delcenserie R, et al.	2001	France	53	28	N/A	Ciprofloxacin for 7 or 21 days	No intervention	High
Dellinger EP, et al.	2007	USA	50 (36% females, range 18-64 years (68%), range 65-74 years (18%), >75 years (14%))	50 (24% females, range 18-64 years (68%), range 65-74 years (18%), >75 years (14%))	Biliary (44% vs. 24%), alcohol (36% vs. 52%), other (20% vs. 24%)	Meropenem 1 g i.v. every 8 h for a minimum of 7 days, and a maximum of 21 days, with a recommended duration of 14 days	Dose- and administration-matched placebo	High
Finch WT, et al.	1976	USA	31 (39% females, mean age = 34.6 years, range 19-58 years)	27 (44% females, mean age = 36.9 years, range 15-87 years)	Alcohol (71% vs. 59%), biliary (6% vs. 8%), undetermined (23% vs. 33%)	Ampicillin 500 mg i.v. every 6 h, or ampicillin 1 g i.v. every 6 h, or cephalothin 1 g i.v. every 6 h for 7 days	No intervention	High
Garcia-Barrasa A, et al.	2009	Spain	22 (36% females, mean age = 59.5 years, range 31-84 years)	19 (21% females, mean age = 67 years, range 38-79 years)	Biliary (72.7% vs. 57.9%), alcohol (9.1% vs. 26.3%), others (18.2% vs. 15.8%)	Ciprofloxacin 300 mg i.v. every 12 h for 10 days	Identical placebo	High
Hejtmankova S, et al.	2003	Czech Republic	20	21	N/A	Meropenem 500 mg i.v. every 8 h for 10 days	No intervention	High
Hubaczova M, et al.	2001	Czech Republic	33	30	N/A	Metronidazole 500 mg i.v. every 8 h, ciprofloxacin 200 mg i.v. every 12 h	No intervention	High
Isenmann R, et al.	2004	Germany	58 (26% females, median age = 47.9 years, range 25.1-72.5 years)	56 (21% females, median age = 45.6 years, range 21.9-78.4 years)	Alcohol (55% vs. 60%), biliary (22% vs. 16%), other (22% vs. 24%)	Ciprofloxacin 400 mg i.v. every 12 h, metronidazole 500 mg i.v. every 12 h for 21 days	Placebo	High

Llukacaj A, et al.	2003	Albania	40	40	N/A	Imipenem 750 mg i.v. every 12 h for 7 days	Placebo	High
Luiten EJT, et al.	1995	The Netherlands	50 (38% females, mean age = 56 years, range 26-91 years)	52 (44% females, mean age = 55 years, range 20-88 years)	Alcohol (38% vs. 23%), gallstones (34% vs. 37%), hyperparathyroidism (0% vs 4%), blunt abdominal trauma (2% vs. 0%), postoperative (4% vs. 4%), ERCP-induced (2% vs. 5%), unknown (20% vs. 27%)	Colistin sulfate 200 mg orally every 6h, amphotericin 500 mg orally every 6 h, norfloxacin 50 mg orally every 6 h plus the aformentioned daily dose was also given in a rectal enema every day and cefotaxime sodium 500 mg i.v. every 8 hours	No intervention	High
Nordback I, et al.	2001	Finland	25 (8% females, mean age ± SD = 47±8 years)	33 (15% females, mean age ± SD = 46±7 years)	Alcohol (80% vs. 76%), biliary (4% vs. 6%), other (16% vs. 18%)	Imipenem cilastatin 1 g i.v. every 8 h for two weeks	No intervention	High
Pederzoli P, et al.	1993	Italy	41 (mean age = 54 years)	33 (mean age = 50 years)	N/A	Imipenem 500 mg i.v. every 8 hours for 14 days	No intervention	High
Poropat G, et al.	2019	Croatia	49 (41% females, median age = 74, range 43-88 years)	49 (45% females, median age = 74, range 46-93 years)	Biliary (61% vs. 59%), alcohol 25% vs. 23%, hypertriglyceridemia (4% vs. 4%), Post-ERCP (4% vs. 6%), other (6% vs. 8%)	Imipenem-cilastatin 500 mg i.v. every 8 hours for ideally 10 days, a minimum of 7 and a maximum of 21 days	Placebo	Low
Qu R, et al.	2012	China	36 (29% females, mean age ± SD = 43.21±11.12 years)	35 (28% females, mean age ± SD = 43.72±10.98 years)	N/A	Antibiotic for 14 days	No intervention	High
Rokke O, et al.	2007	Norway	36 (36% females, median age = 60, range 19-90 years)	37 (30% females, median age = 57, range 29-84 years)	N/A	Imipenem 500 mg i.v. every 8 hours for 5-7 days	No intervention	High
Sainio V, et al.	1995	Finland	30 (10% females, mean age ± SD = 43±11.3 years)	30 (13% females, mean age ± SD = 38.7 ± 8.4 years)	All patients with alcohol-induced pancreatitis	Cefuroxime 1.5 g i.v. every 8 h until clinical recovery and fall to normal of CRP concentrations	No intervention	High

Schwarz M, et al.	1997	Germany	13<br>(median age = 43, range 31-82)	13<br>(median age = 46, range 24-71)	N/A	Ofloxacin 2 x 200 mg i.v. daily and Metronidazol 2 x 500 mg i.v. daily for 10 days	No intervention	High
Spicak J, et al.	2004	Czech Republic	17	18	N/A	Ciprofloxacin 2 x 200 mg and metronidazol 3 x 500 mg i.v. daily or meropenem 3 x 500 mg i.v. daily for 10 days	No intervention	High
Xue P, et al.	2009	China	29<br>(52% females, mean age ± SD = 48.4 ± 15.1 years)	27<br>(48% females, mean age ± SD = 47.5 ± 12.3 years)	Biliary (53.6% vs. 53.8%), alcohol (14.3% vs. 7.2%), Hyperlipidemia (7.1% vs. 7.7%), Idiopathic (27.6% vs. 33.3%)	Imipenem-cilastatin 500 mg i.v. every 8 hours for 7-14 days	No intervention	High
Yang XN, et al.	2009	China	26 (46% females, mean age ± SD = 47.5±12.3 years)	28 (54% females, mean age ± SD = 48.4±15.1 years)	N/A	Imipenem-cilastatin 0.5 g i.v. every 8 hours for 10 days	No intervention	High
